# CSF sTREM2 in delirium—relation to Alzheimer’s disease CSF biomarkers Aβ42, t-tau and p-tau

**DOI:** 10.1186/s12974-018-1331-1

**Published:** 2018-11-03

**Authors:** Kristi Henjum, Else Quist-Paulsen, Henrik Zetterberg, Kaj Blennow, Lars N. G. Nilsson, Leiv Otto Watne

**Affiliations:** 1 Department of Pharmacology, Institute of Clinical Medicine, University of Oslo and Oslo University Hospital, P.O. box 1057 Blindern, 0316 Oslo, Norway; 2 Department of Infectious Diseases, Oslo University Hospital, Ullevaal Hospital, P.O. Box 4956 Nydalen, N-0450 Oslo, Norway; 3 Institute of Clinical Medicine, University of Oslo, P.O. Box 1171 Blindern, 0318 Oslo, Norway; 4 Department of Psychiatry and Neurochemistry, Institute of Neuroscience and Physiology, The Sahlgrenska Academy at University of Gothenburg, SE-431 80 Mölndal, Sweden; 5 Clinical Neurochemistry Laboratory, Sahlgrenska University Hospital, SE-431 80 Mölndal, Sweden; 6 Department of Degenerative Disease, UCL Institute of Neurology, Queen Square, Gower Street, London, WC1E 6BT UK; 7 UK Dementia Research Institute at UCL, Gower Street, London, WC1E 6BT UK; 8 Oslo Delirium Research Group, Department of Geriatric Medicine, Oslo University Hospital, PO box 4950 Nydalen, N-0424 Oslo, Norway; 9 Institute of Basic Medical Sciences, University of Oslo, Domus Medica, Sognsvannsveien 9, N-0372 Oslo, Norway

**Keywords:** Delirium, Dementia, Alzheimer’s disease, CSF biomarkers, Soluble TREM2

## Abstract

**Background:**

Delirium and dementia share symptoms of cognitive dysfunctions, and mechanisms of neuroinflammation appear involved in both conditions. Triggering receptor expressed on myeloid cells 2 (*TREM2*) is linked to dementia and neurodegenerative disease. It encodes expression of an innate immune receptor in the brain expressed by microglia. The level of the soluble fragment of TREM2 (sTREM2) is reported to increase in the cerebrospinal fluid (CSF) already in prodromal and asymptomatic Alzheimer’s disease.

**Methods:**

We analyzed the level of CSF sTREM2 in relation to delirium and dementia. The study included patients with or without pre-existing dementia who underwent acute hip fracture surgery (*n* = 120), and some of the patients developed delirium (*n* = 65). A medical delirium cohort (*n* = 26) was also examined. ELISA was used to determine the level of sTREM2 in CSF.

**Results:**

Delirium was associated with a higher level of CSF sTREM2 only among those without pre-existing dementia (*p* = 0.046, *n* = 15, *n* = 44), particularly among patients developing delirium after CSF sampling (*p* = 0.02, *n* = 7, *n* = 44). Between patients with dementia, there was no group difference, but the CSF sTREM2 level increased with waiting time for surgery (*r*_S_ = 0.39, *p* = 0.002, *n* = 60) and correlated well with the CSF Alzheimer’s disease biomarkers, Aβ42, and t-tau/p-tau (*r*_S_ = 0.40, *p* = 0.002, *r*_S_ = 0.46, *p* < 0.001/ *r*_S_ = 0.49, *p* < 0.001, *n* = 60). Among patients with dementia, the level of Aβ38 and Aβ40 also correlated positively with sTREM2 in CSF (Aβ38_MSD_*r*_S_ = 0.44, *p* = 0.001; Aβ40_MSD_*r*_S_ = 0.48, *p* < 0.001; Aβ42_MSD_*r*_S_ = 0.43, *p* < 0.001, *n* = 60).

**Conclusion:**

The findings reinforce the involvement of neuroinflammation in delirium, yet with separate responses in patients with or without pre-existing dementia. Our findings support the concept of primed microglia in neurodegenerative disease and central immune activation after a peripheral trauma in such patients. A CSF biomarker panel of neuroinflammation might be valuable to prevent delirium by identifying patients at risk.

**Electronic supplementary material:**

The online version of this article (10.1186/s12974-018-1331-1) contains supplementary material, which is available to authorized users.

## Background

Delirium is an acute state of confusion with fluctuating symptoms of disturbed attention and cognition commonly precipitated by stress, such as surgery, in frail patients [1]. Besides unpleasant while ongoing, a delirium carries the risk of increased mortality and long-term sequela of cognitive functions [2]. Although much is unknown, the delirium pathogenesis is thought to involve disturbed neurotransmission and/or induced inflammation with microglial activation [3]. The neuroinflammatory hypothesis suggest that delirium symptoms arise as central immunity is activated by initial peripheral inflammation that convey to the brain [4, 5].

While delirium increases the risk of dementia, dementia is also a delirium risk factor [6–8]. The most common cause of dementia is Alzheimer’s disease (AD), a neurodegenerative disorder with pathological hallmarks amyloid plaques and neurofibrillary tangles [9]. Already at a preclinical stage with evident neuropathology is AD found to increase the risk of delirium [10, 11]. Neuroinflammation with microglial activation and astrogliosis also plays a role in AD [12]. Thus, delirium and dementia etiology are intertwined with shared pathogenic mechanisms such as microglial activation and other facets of neuroinflammation [13].

**Fig. 1:**
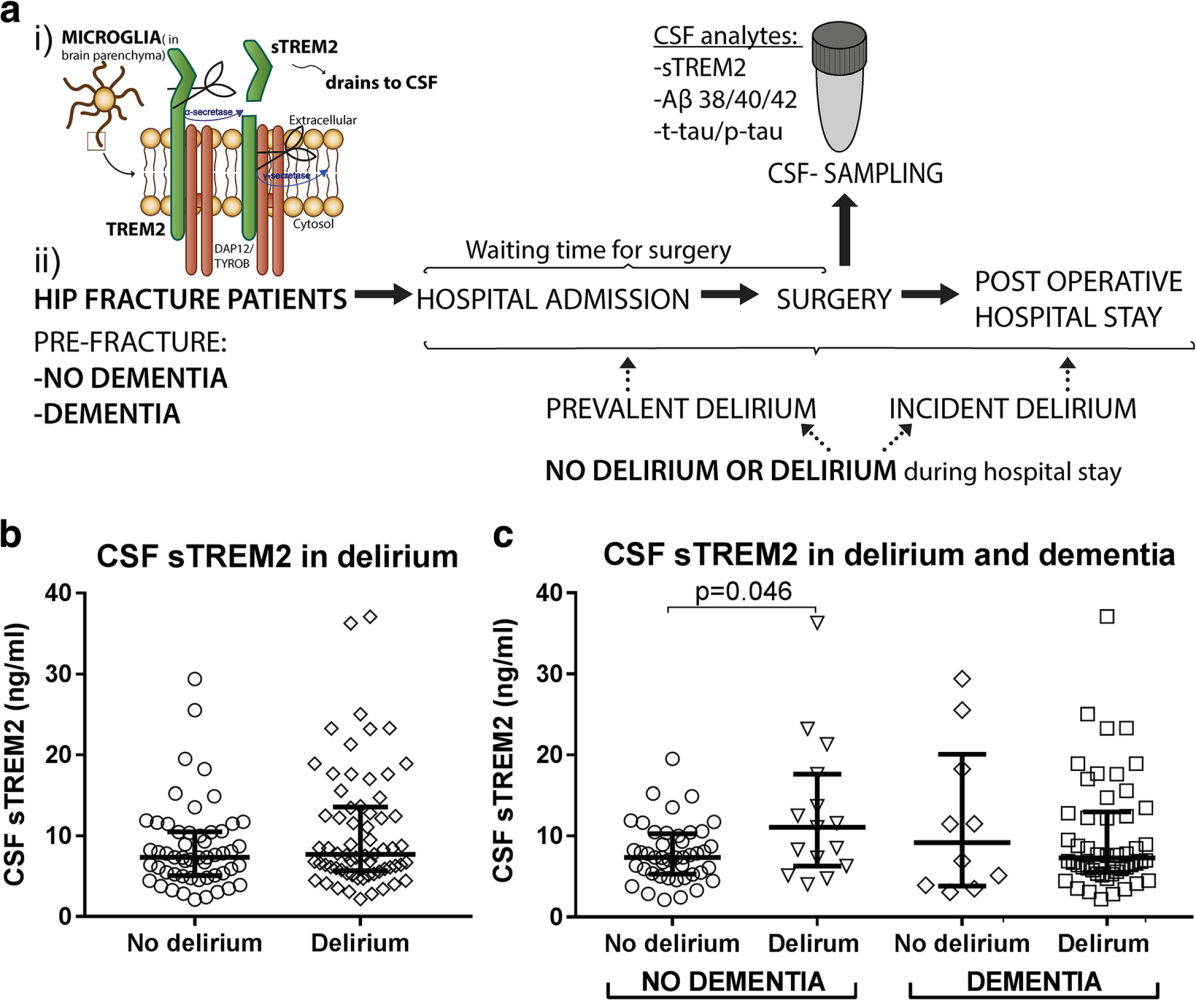
CSF sTREM2 in patients hospitalized by a hip fracture. **a** A fragment of the microglial receptor TREM2 and sTREM2, released after ectodomain shedding, drains to the CSF. Sampling and time line of hip fracture patients admitted to the hospital where some of them developed delirium. **b** CSF sTREM2 level did not discriminate patients not developing delirium from patients developing delirium during hospitalization for an acute hip fracture surgery (*p* = 0.25, *n* = 54, *n* = 65). **c** Stratification and separate analyses of patients with and without pre-existing dementia showed significantly higher CSF sTREM2 level in delirium of patients without pre-existing dementia (*p* = 0.046, *n* = 15, *n* = 44). Dementia patients with and without delirium had a similar CSF sTREM2 level (*p* = 0.94, *n* = 10, *n* = 50). Two tailed *p* values were obtained by Mann-Whitney test, while larger and smaller lines represent the median and interquartile range respectively. CSF: cerebrospinal fluid, sTREM2: soluble TREM2

Microglia, the resident immune cells of the brain, express the innate immune receptor triggering receptor expressed on myeloid cells 2 (TREM2) [14]. Variants of *TREM2* are known as dementia risk factors [15–17] linking *TREM2* to age-related neurodegeneration. The transmembrane TREM2 receptor undergoes ectodomain shedding releasing soluble TREM2 (sTREM2) [18] (Fig. 1a). In the cerebrospinal fluid (CSF) of AD patients, sTREM2 is reported increased [19, 20]. An even higher level is observed at the prodromal mild cognitive impairment (MCI) stage of AD [21]. Moreover, the level of CSF sTREM2 correlates positively with the core CSF biomarkers amyloid beta 1–42 (Aβ42), total-tau (t-tau), and phosphorylated-tau (p-tau) in asymptomatic patients, which further suggests an early involvement of reactive microgliosis [22, 23].

In the present study, we analyzed the CSF sTREM2 level in patients with or without pre-existing dementia. The patients all suffered a hip fracture with subsequent hospital admission and surgery that for some led to delirium, and we evaluated CSF sTREM2 as a putative biomarker of delirium. Given the abovementioned biomarker correlations in AD, we also examined the relation between CSF sTREM2 and AD core biomarkers, CSF Aβ42, t-tau, and p-tau. For the potential influence of a peripheral trauma, we investigated how the CSF sTREM2 level related to time after hip fracture. We also included a patient group with delirium associated with a medical condition to evaluate potential similarities and dissimilarities to hip fracture-triggered delirium.

## Methods

### Hip fracture cohort

The hip fracture patients, which were recruited from the Oslo Orthogeriatric trial (OOT), were admitted to the Oslo University Hospital Ullevål (OUS, Ullevål) between September 2009 and January 2012 [24, 25]. Delirium was assessed using the Confusion Assessment Method (CAM) [26] by the study physician or a study nurse. Delirium was assessed daily preoperatively and until the fifth postoperative day or in case of delirium until discharge. Pre-fracture dementia status was decided by consensus and based on the International Classification of Diseases − 10 (ICD-10) criteria for dementia by an expert panel as previously described [25].

The hip fracture patients (*n* = 120) were grouped both according to delirium and dementia status into either of the following groups (see Fig. 1a and Table 1)

1. No delirium during the hospital stay (no delirium) (*n* = 54)
  - No delirium, and without pre-fracture dementia (*n* = 44)
  - No delirium, but pre-fracture dementia (*n* = 10)
2. Delirium during the hospital stay (delirium) (*n* = 65)
  - Delirium, but without pre-fracture dementia (*n* = 15)
  - Delirium with pre-fracture to dementia (*n* = 50)

Delirium patients included prevalent delirium (those that developed delirium preoperatively; *n* = 41) and incident delirium (postoperative delirium in those free from delirium before surgery; *n* = 21). The sub-classification of delirium onset was applied for delirium onset analyses.

### Medical delirium cohort

The medical delirium cohort was recruited from a prospective study at the same hospital in which 244 patients who underwent lumbar puncture (LP) due to suspicion of acute central nervous system (CNS) infection were included. Patients were included between January 2014 and December 2015. The patients included in the current study (*n* = 26) were those in which a CNS infection was ruled out and delirium triggered by another medical condition was considered the most likely explanation for the acute cognitive symptoms. Pneumonia and urinary tract infection were the most frequent diagnoses in this group. All patients had encephalopathy at the time of the LP. Delirium was assessed either by the study physician with CAM, or by clinical evaluation of treating physician in the medical ward. Dementia status was set from the hospital records. These delirium patients formed a separate group labeled “medical delirium” afflicted by delirium with another precipitating factor than the hip fracture patients.

### CSF sampling, handling, and storage

In the hip fracture cohort, CSF was collected in connection with the orthopedic surgery at the onset of spinal anesthesia before administrating the anesthetic agents. CSF of patients with medical delirium was obtained during the diagnostic lumbar puncture (LP) at a median of 1 day after CNS symptoms developed. CSF was collected in polypropylene tubes and centrifuged as soon as possible, and supernatant aliquots were stored in polypropylene tubes at − 80 °C [27].

**Table 1:** Characteristics of the hip fracture and medical delirium patients

	Hip fracture cohort					Medical delirium
	All	No delirium	Delirium			
			All	Incident	Prevalent	Medical
**PATIENTS** ***WITHOUT*** **DEMENTIA**						
*N*	*59*	*44*	*15*	*7*	*8*	*17*
Age (years)	84 (10)	84 (16)	85 (7)	86 (11)	85 (7)	66 (16)
Gender						
Male	17	11	6	3	3	10
Female	42	33	9	4	5	7
Time to surgery (h)^*^	23 (17)	23 (17)	27 (15)	22 (19)	30 (14)	–
CSF sTREM2 ng/ml	7.8 (5.7)	7.4 (5)	11.1(11)	11.6 (5)	7.8 (15)	5.6 (8)
CSF biomarkers						
*N*	*57*	*44*	*13*	*5*	*8*	
CSF Aβ42 (pg/ml)	446 (367)	479 (414)	283 (224)	283 (232)	295 (253)	–
CSF t-tau (pg/ml)	369 (276)	356 (198)	564 (638)	564 (369)	587 (795)	–
CSF p-tau (pg/ml)	57(35)	54 (33)	78 (68)	78 (19)	88 (89)	–
CSF Aβ42 cut-off (< 530 pg/ml)						
Below	37	26	11	4	7	–
Above	20	18	2	1	1	–
CSF p-tau cut-off (≥ 60 pg/ml)						
Above	26	17	9	4	5	–
Below	31	27	4	1	3	–
CSF t-tau cut-off (> 350 pg/ml)						
Above	32	23	9	5	4	–
Below	25	21	4	–	4	–
**PATIENTS** ***WITH*** **DEMENTIA**						
*N*	*61*	*10*	*50*	*13*	*33*	*9*
Age (years)	86 (9)	86.5 (17)	85(9)	87 (8)	85 (8)	71(23)
Gender						
Male	16	1	15	5	8	6
Female	45	9	35	8	25	3
Time to surgery (h)^*^	26 (28)	27 (16)	26(31)	18(19)	38 (24)	–
CSF sTREM2 ng/ml	7.0 (7.8)	9.2 (16)	7.3 (7)	6.1 (10)	8.5 (8)	6.9(6.7)
CSF biomarkers, *N*	*60*	*9*	*50*	*13*	*33*	
CSF Aβ42 (pg/ml)	265 (166)	317 (290)	258 (172)	268 (219)	269 (174)	–
CSF t-tau (pg/ml)	408 (379)	441 (503)	408 (366)	385 (293)	407 (329)	–
CSF p-tau (pg/ml	55 (41)	58 (57)	55 (35)	55 (47)	55 (31)	–
CSF Aβ42 cut-off (< 530 pg/ml)						
Below	55	7	47	11	1	–
Above	5	2	3	2	32	–
CSF p-tau cut-off (≥ 60 pg/ml)						
Above	24	4	20	5	13	–
Below	36	5	30	8	20	–
CSF t-tau cut-off (> 350 pg/ml)						
Above	38	5	33	8	22	–
Below	22	4	17	5	11	–

Data are presented as median and interquartile range (IQR), waiting time for surgery. Aβ42, amyloid beta 1–42; t-tau, total-tau; p-tau, phosphorylated tau; sTREM2, soluble triggering receptor expressed on myeloid cells

^*^Time to surgery, hours from hospital admission to surgery (onset of anesthesia) and CSF sampling

### CSF sTREM2 measurements

CSF sTREM2 was assayed by a sensitive TREM2 enzyme-linked immunosorbent assay (ELISA) as previously described [22]. Briefly, plates were incubated with an anti-humanTREM2 polyclonal capture antibody overnight at 4 °C (AF1828, R&D Systems, Minneapolis, MN, USA) and TREM2 detected by a mouse anti-human TREM2 monoclonal HRP-conjugated antibody (1 h incubation at room temperature (RT);SEK11084, Sino Biologics, Beijing, China). Samples were assayed in duplicates (2 h incubation at RT) with known cohort (hip fracture or medical), but with the clinical identity unknown to the operator. Samples with extreme values were assayed again, including the same and an increased sample dilution to verify measurements in the repeated assay. Two internal standard (CSF) samples were included in each assay to assess interday variability and used to adjust the medical delirium cohort which was assayed separately from the hip fracture cohort, with a final CV < 10% across assays.

### CSF Aβ, t-tau, and p-tau measurements

CSF levels of t-tau, p-tau, and Aβ42 were quantified with commercially available ELISAs; Innotest® hTau Ag, Innotest® phoshoTau (181P), and Innotest® β-amyloid 1–42 as previously described [28–30] (Fujirebio Europe, Gent, Belgium). CSF Aβ peptide levels were determined with CSF Aβ_1–38_ (Aβ38), Aβ_1–40_ (Aβ40), and Aβ_1–42_ (Aβ42) MSD Triplex assay (Meso Scale Discovery, Rockwilly, MA, USA). All these analyses were performed at the Clinical Neurochemistry Laboratory at Sahlgrenska University Hopsital, Mölndal, Sweden. CSF cut-off for pathological level was < 530 pg/ml (Aβ42+/−), ≥ 60 pg/ml (p-tau+/−), and > 350 pg/ml (t-tau +/−) [31].

### Statistical analyses

Analyses were performed by parametric or non-parametric statistics as appropriate depending on the data distribution. Data distribution was assessed by histogram, probability-probability (P-P), and quantile-quantile (Q-Q) plots. As CSF sTREM2 raw data were skewed, continuous data are reported by median (interquartile range (IQR)) and group differences analyzed by Mann-Whitney or Kruskal-Wallis test. *p* values of group comparisons were obtained by Mann-Whitney test, unless otherwise reported. The correlation analyses are reported by Spearman’s rho correlation coefficient (*r*_*s*_).

Multiple linear regression was used for analyses with multiple predictor variables. For stepwise multiple linear regression, predictors were included based on significance in univariate analyses and biological grounds, including predictors with highest assumed degree of explained variability first. The data transformation by the natural logarithm (ln) (ln(CSF sTREM2 ng/ml) approximated a normal distribution and was therefore applied for analyses requiring parametric tests (linear regressions). Standardized residuals in linear regressions met the criteria of normal distribution. In regression analyses with delirium, no delirium was coded as 0 and delirium as 1. In linear regressions with delirium onset; no delirium was coded as 0, incident delirium as 1, and prevalent delirium as 2.

All hypotheses were two-sided and the reported *p* values are therefore two-tailed. The significance level was set at *p* < 0.05. Statistical analyses were performed by the Statistical Package for Social Sciences (SPSS, versions 24 and 25; IBM, Armonk, NY, USA). Graphical illustrations were created with GraphPad Prism (version 7.04 Graph Pad Software, La Jolla, CA, USA).

## Results

### Increased CSF sTREM2 with delirium in hip fracture patients without pre-existing dementia

The patients studied did or did not develop delirium during hospitalization after a hip fracture requiring acute surgery. Around half of the patients included were demented before the accident, and a large proportion of those demented patients developed delirium after the hip fracture (≈ 80%; study setup in Fig. 1a). All the patients displayed an advanced age irrespective of dementia diagnosis (median age 85 years). The gender distribution was similar between groups (Table 1). Gender did not influence the CSF sTREM2 level and was therefore not included in the further analyses (data not shown).

Initial analyses of the CSF sTREM2 level showed a considerable intragroup variability, both among patients with and without delirium, with no separation between these two groups (*p* = 0.25, CSF sTREM2: 7.7(7.9) versus 7.4(5.4) ng/ml, *n* = 65, *n* = 54; Fig. 1b). Neuropathology of several dementia disorders involves microglial activation, and the underlying pathogenic processes may be different in delirium superimposed on dementia as compared to delirium in the absence of pre-existing dementia. Interestingly when stratifying patients according to pre-fracture dementia, an increased CSF sTREM2 level was only associated with delirium among those patients who were not demented before the hip fracture (*p* = 0.046, CSF sTREM2: 11.1(11.0) versus 7.4(5.0) ng/ml, *n* = 15, *n* = 44). In contrast, among patients with a pre-existing dementia, the CSF sTREM2 level did not differ between those patients who did or did not develop delirium (*p* = 0.94, *n* = 50, *n* = 10; Fig. 1c and Table 1).

### CSF sTREM2 in relation to onset of delirium in hip fracture patients

**Fig. 2:**
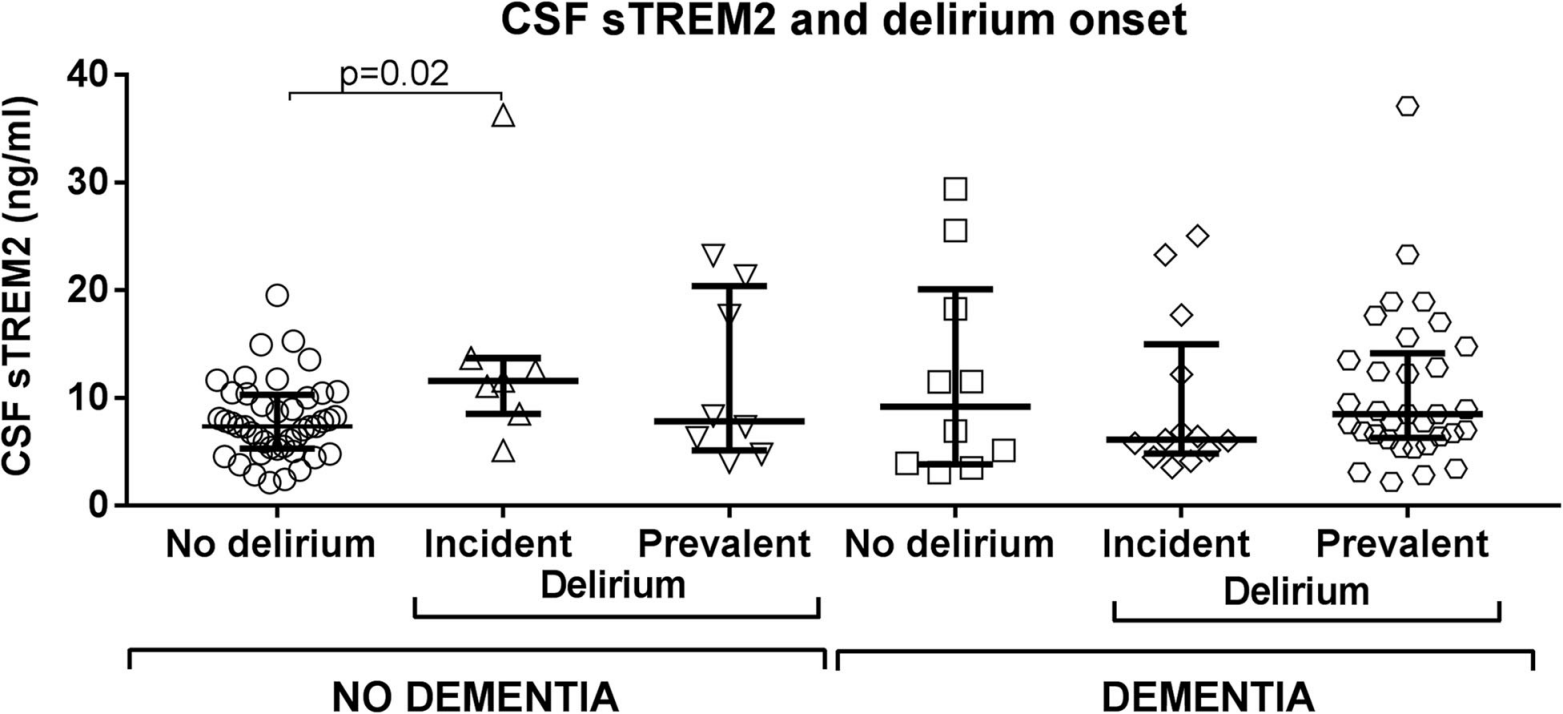
CSF sTREM2 in delirium separated by onset in patients with and without dementia. Delirium patients were separated by symptom onset prior to or after hip fracture surgery (prevalent- and incident delirium respectively). Among patients without dementia, patients with delirium after CSF sampling (incident delirium) displayed a significantly higher CSF sTREM2 level than patients without delirium (*p* = 0.02, *n* = 7, *n* = 44). CSF sTREM2 in patients with delirium before CSF sampling (prevalent delirium) did not differ from neither those with incident delirium nor from patients without delirium (*p* = 0.45 and *p* = 0.46, *n* = 7, *n* = 8, *n* = 44). Separating delirium patients with dementia by delirium onset before or after surgery did not reveal any differences between patient groups. The *p* values are two-tailed and obtained by Mann-Whitney test, larger and smaller lines represent the median and interquartile range respectively. CSF: cerebrospinal fluid, sTREM2: soluble TREM2

During delirium, microglial reactivity may be transient and CSF sTREM2 levels may therefore fluctuate in a manner not directly related to clinical symptoms. To investigate possible changes in CSF sTREM2 with delirium progression, we stratified the delirium patients by onset of delirium relative to time of surgery when CSF was sampled (see Fig. 1a). Patients with pre-operational delirium were classified as having prevalent delirium, while those with post-operational delirium were categorized as having incident delirium. Among patients without pre-existing dementia, the incident delirium group showed an increased CSF sTREM2 level as compared to patients not developing delirium (*p* = 0.02, CSF sTREM2: 11.6 (5.0) versus 7.4 (5.0) ng/ml, *n* = 7, *n* = 44). The patients with prevalent delirium displayed a large variability in CSF sTREM2 and did not differ statistically neither from the unaffected patient group nor from the group having incident delirium (*p* = 0.46, *p* = 0.45, *n* = 8, *n* = 44, *n* = 7). When likewise stratifying patients with pre-existing dementia into subgroups of onset of delirium, the CSF sTREM2 level did not differ between the three groups (*p* = 0.62, Kruskal-Wallis test, Fig. 2 and Table 1).

### CSF sTREM2 in relation to waiting time for surgery among hip fracture patients

**Fig. 3:**
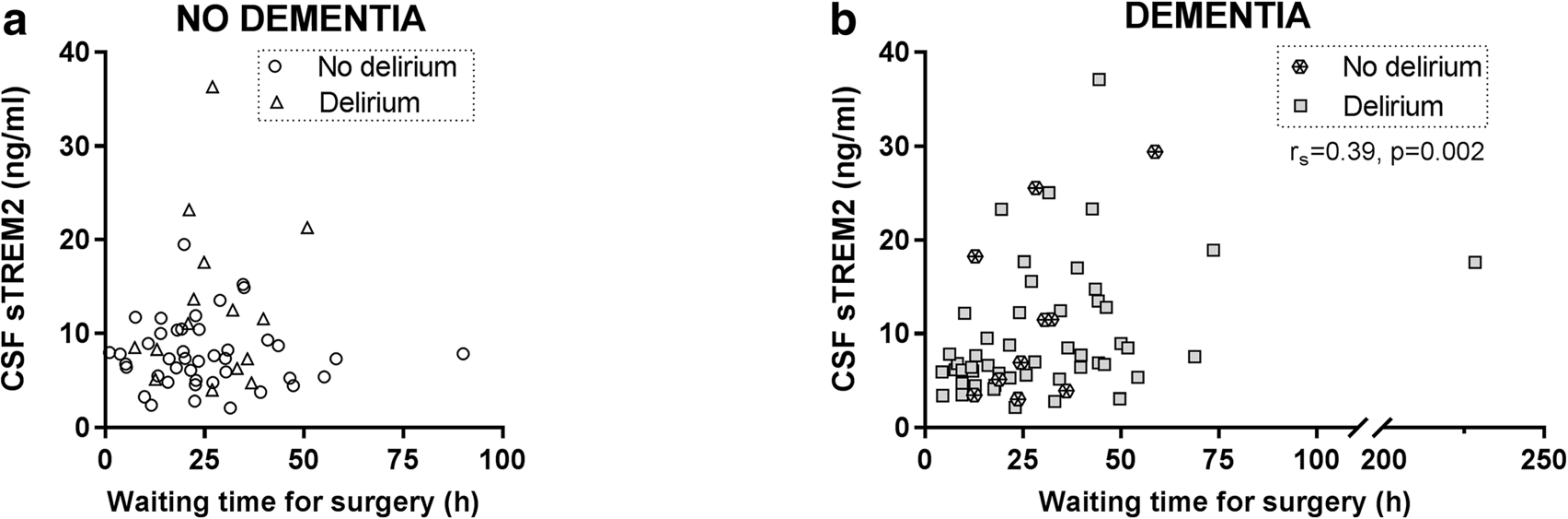
Time-dependent influence of waiting time for hip fracture surgery the CSF sTREM2. CSF was sampled immediately before surgery started. **a** Among patients without dementia, there was no correlation between the CSF sTREM2 level and waiting time for surgery from hospital admission (hours; waiting time for surgery). **b** In contrast, among dementia cases, the CSF sTREM2 level increased with time from hospital admission until surgery with a positive correlation coefficient (*r*_s_). The correlations are calculated for all patients with or without pre-existing dementia respectively, but patients with and without delirium are indicated by separate symbols. CSF: cerebrospinal fluid, sTREM2: soluble TREM2. *r*_s_: Spearman’s rho

A peripheral insult, such as a hip fracture, may trigger a central immune response [32]. The CSF sTREM2 level correlated positively to waiting time for surgery after hospital admission (waiting time for surgery (h); *r*_S_ = 0.23, *p* = 0.01, *n* = 119). The positive correlation in all patients was presumably due to a stronger positive relationship between CSF sTREM2 and waiting time for surgery among patients with pre-existing dementia, which remained when demented patients not developing delirium were excluded (*r*_S_ = 0.39, *p* = 0.002, *n* = 60; *r*_S_ = 0.40, *p* = 0.005, *n* = 49, Fig. 3 and Table 2).

**Table 2:** CSF sTREM2 Spearman’s Rho correlations in the hip fracture cohort

	PATIENTS *WITHOUT* DEMENTIA						PATIENTS *WITH* DEMENTIA					
	All		No delirium		Delirium		All		No delirium		Delirium	
N	*59*		*44*		*15*		*61 (60)*		*10*		*50*	
	Rho	p	Rho	p	Rho	p	Rho	p	Rho	p	Rho	p
Age	0.21	0.12	0.12	0.43	0.23	0.40	0.00	0.98	0.16	0.66	-0.05	0.74
Time to surgery (hours) *	0.01	0.93	-0.03	0.83	0.06	0.84	0.39	0.002	0.43	0.21	0.40	0.005
CSF Biomarkers												
N	*57*		*44*		*13*		*60*		*9*		*50*	
	Rho	p	Rho	p	Rho	p	Rho	p	Rho	p	Rho	p
CSF Aβ42 (pg/ml)	0.11	0.40	0.20	0.18	0.31	0.30	0.40	0.002	0.02	0.97	0.53	<0.001
CSF Aβ38 (pg/ml) MSD	0.09	0.49	0.06	0.71	0.18	0.55	0.44	<0.001	0.40	0.29	0.45	0.001
CSF Aβ40 (pg/ml) MSD	0.14	0.32	0.12	0.44	0.23	0.45	0.48	<0.001	0.37	0.33	0.51	<0.001
CSF Aβ42 (pg/ml) MSD	0.09	0.52	0.24	0.12	-0.13	0.67	0.43	0.001	0.15	0.70	0.53	<0.001
CSF t-tau (pg/ml)	0.14	0.28	-0.04	0.82	0.20	0.51	0.46	<0.001	0.83	0.005	0.34	0.016
CSF p-tau (pg/ml)	0.12	0.38	-0.02	0.90	0.23	0.46	0.49	<0.001	0.77	0.016	0.37	0.008

Aβ42, amyloid beta 1–42; t-tau, total-tau; p-tau, phosphorylated tau; sTREM2, soluble triggering receptor expressed on myeloid cells

*Time to surgery, hours from hospital admission to surgery (onset of anesthesia) and CSF sampling

Having found CSF sTREM2 to relate positively to surgery waiting time among patients with pre-existing dementia, we were concerned that this masked an effect of delirium on CSF TREM2 in our previous analyses of patients with pre-existing dementia. We adjusted for surgery waiting time, but delirium did still not affect the CSF TREM2 level in this group of demented patients (multiple linear regression bivariate model; waiting time for surgery (h): *β*1 = 0.007, *p* = 0.02, delirium: *β*2 = − 0.12, *p* = 0.60, *n* = 59, Table 3). The same analyses of delirium patients with pre-existing dementia sub-grouped relative to delirium onset (incident or prevalent delirium) reiterated that surgery waiting time, but not delirium onset, influenced the CSF TREM2 level (data not shown).

**Table 3:** Influence of multiple predictors on CSF sTREM2^a^ analyzed by multiple linear regression

Patient group	Analysis	Predictor		
		β	p	n
Dementia	Delirium adjusted by waiting time for surgery			59
	Waiting time for surgery (β1)	0.007	0.02	
	Delirium (β2)	-0.12	0.60	
No dementia	Delirium (at any time)			59
	Delirium (univariate)	0.40	0.01	
	Delirium (adjusted) (β1)	0.35	0.03	
	Age (β2)	0.01	0.22	
	Delirium onset: incident delirium			51
	Incident delirium (univariate)	0.52	0.01	
	Incident delirium (β1)	0.47	0.03	
	Age (β2)	0.01	0.26	
Dementia	AD pathological CSF-biomarkers, age and delirium			59
	p-tau (β1)	0.01	<0.001	
	Aβ42 (β2)	0.002	0.002	
	Age (β3)	0.0001	0.91	
	Delirium (β4)	- 0.03	0.86	
No dementia	AD pathological CSF-biomarkers, age and delirium			57
	Delirium (β1)	0.47	0.02	
	p-tau (β2)	0.0000	0.97	
	Aβ42 (β3)	0.0000	0.16	
	Age (β4)	0.013	0.13	
No dementia	Incident (hip fracture) and medical delirium age adjusted			24
	Incident delirium (univariate)	0.62	0.05	
	Incident delirium (β1)	0.27	0.48	
	Age (β2)	0.02	0.15	

^a^*Linear regression with the dependent variable ln (sTREM2ng/ml)*. Aβ42; amyloid beta 1-42, t-tau; total-tau; p-tau; *phosphorylated tau, sTREM2; soluble triggering receptor expressed on myeloid cells*

### CSF sTREM2 in relation to age among hip fracture patients

The study group was of an advanced age (median of 85 years), and CSF sTREM2 did not relate to patient age (*r*_S_ = 0.09, *p* = 0.32, *n* = 120, Additional file 1: Figure S1). Morbidity or comorbidity might have masked an effect of aging on CSF sTREM2 that we observed in a cohort with a wider age distribution [22]. However, CSF sTREM2 did still not relate to age when restricting the analysis to the patient group having neither pre-existing dementia nor delirium (*r*_S_ = 0.12, *p* = 0.43, *n* = 44; Additional file 1: Figure S1A and Table 2). The four study groups of the hip fracture cohort, patients with or without delirium and with or without pre-existing dementia, were age-matched when assessing median age. However, the patient group having neither pre-existing dementia nor delirium had a greater proportion of relatively young individuals than the other groups. Neuroinflammation is a feature of aging, and we wanted to ensure that the age distribution did not infer with our analyses. We approached this by adjusting for age and analyzing data with multiple linear regression models. CSF sTREM2 remained significantly increased with delirium in age-adjusted analyses of patients without dementia (*p* < 0.05, ln(CSFsTREM2); univariate model: delirium *β*1 = 0.40, *p* = 0.01; with a bivariate model: delirium *β*1 = 0.35 *p* = 0.03; age *β*2 = 0.01, *p* = 0.22, *n* = 59). This finding reiterated when reanalyzing the effect of incident delirium among patients without dementia (ln(CSFsTREM2): incident delirium *β*1 = 0.52, *p* = 0.01; a bivariate model: incident delirium *β*1 = 0.47, *p* = 0.03; age *β*2 = 0.01, *p* = 0.26, *n* = 51, Table 3).

### CSF sTREM2 in relation to amyloid-β metabolism and plaque sequestration in hip fracture patients

In several reports, CSF sTREM2 level relates to CSF core biomarkers of Alzheimer’s disease Aβ42, t-tau, and p-tau [19, 21, 22]. We hypothesized observing such relations in the present study, with the comorbidities delirium or pre-existing dementia acting as possible confounders. First the relations were analyzed in all patients, then patients were stratified by dementia status and finally by delirium. After stratification by both factors, only two patient groups, those neither having pre-existing dementia nor delirium (*n* = 44) and those being afflicted by both conditions simultaneously (*n* = 50) were of a sufficient group size to merit further statistical analyses (Table 1).

**Fig. 4:**
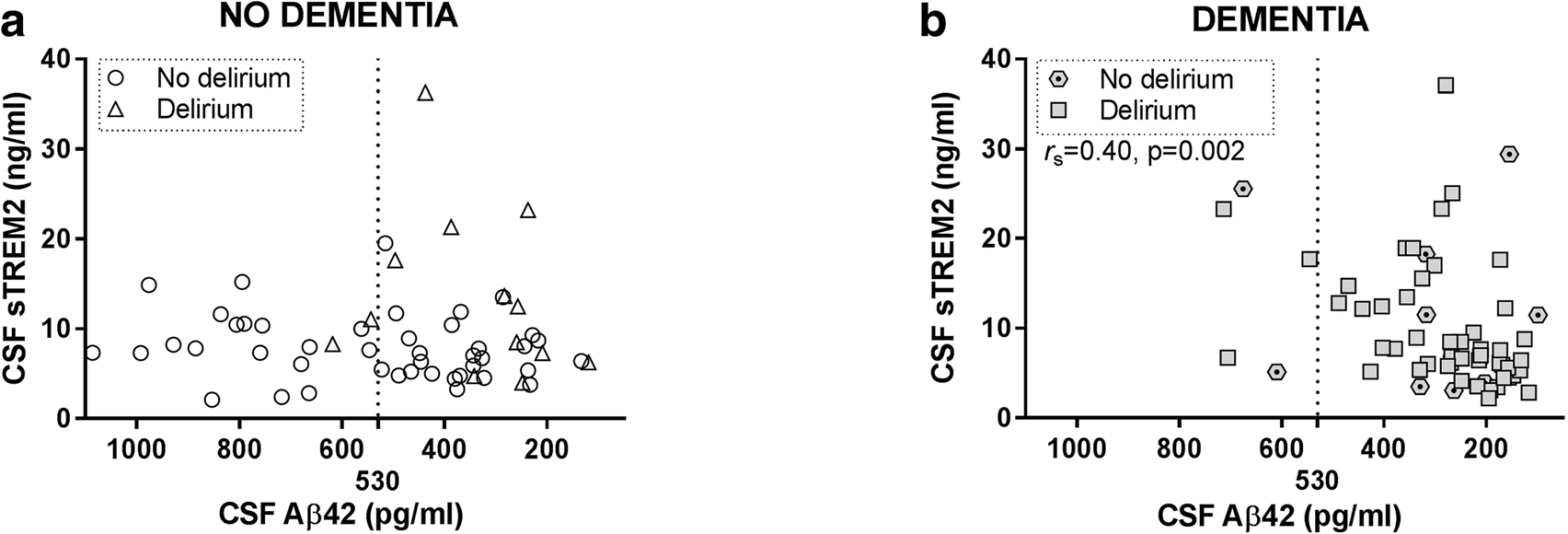
Relation between the level of CSF biomarkers sTREM2 and Aβ42 in hip fracture patients. **a** Among patients without pre-existing dementia, there was no correlation between sTREM2 and Aβ42 in CSF, but (**b**) a positive relation in patients with dementia as indicated by a significant and positive correlation (*r*_s_). The correlations are calculated for all patients with or without dementia respectively, but patients with and without delirium are indicated by separate symbols. The dotted lines show the pathological biomarker cut-off level (530 pg/ml). To present Figs. 4 and 5 in the same manner, the *x*-axes in Fig. 4 are reversed because a low level indicates Aβ pathology. CSF: cerebrospinal fluid, Aβ42: amyloid beta 1–42, sTREM2: soluble TREM2. r_s_: Spearman’s rho

Reduced CSF Aβ42 is assumed to reflect parenchymal Aβ sequestration by plaques in AD brain. CSF sTREM2 and CSF Aβ42 related positively in the entire hip fracture cohort (*r*_S_ = 0.22, *p* = 0.02, *n* = 117). When stratifying study subjects by pre-existing dementia status, CSF sTREM2 and CSF Aβ42 did not correlate in patients without pre-existing dementia, not even after excluding patients with delirium. In contrast, there was a strong positive relation between CSF sTREM2 and CSF Aβ42 of patients with pre-existing dementia which grew stronger when demented patients without delirium were excluded from the analysis (*r*_S_ = 0.40, *p* = 0.002, *n* = 60; *r*_S_ = 0.53, *p* < 0.001, *n* = 50, Fig. 4 and Table 2).

How prone an Aβ peptide is to form amyloid fibrils and be sequestered by a pre-existing Aβ deposit in the brain heavily depends on the C-terminal extension. Indeed, in clinical studies, only the level of hydrophobic Aβ peptides extending to position 42 decrease in CSF when Aβ deposits begin to form in brain [33]. Thus, an increased CSF level of a C-terminal truncated Aβ peptide, e.g., CSF Aβ_1–38_ likely reflect Aβ-monomer metabolism (production or catabolism). Here, we related analyses of CSF Aβ38, Aβ40, and Aβ42 MSD ELISA levels to CSF sTREM2. Our intent was to distinguish if a putative relation between CSF sTREM2 and CSF Aβ42 was linked to Aβ-metabolism or Aβ-plaque sequestration. The MSD analyses outcome was reminiscent to the Innotest Aβ42 analysis, with CSF sTREM2 and CSF Aβ relating positively to all three Aβ peptides (Aβ38 _MSD_, Aβ40 _MSD_, and Aβ42 _MSD_). Upon stratification, the effect was prominent among patients with pre-existing dementia and even stronger when further excluding those patients who did not develop delirium (Aβ38_MSD_*r*_S_ = 0.45, *p* = 0.001; Aβ40_MSD_*r*_S_ = 0.51, *p* < 0.001; Aβ42MSD rS = 0.53, *p* < 0.001, *n* = 50; Additional file 1: Figure S2 and Table 2).

### CSF sTREM2 in relation to CSF tau markers in hip fracture patients

**Fig. 5:**
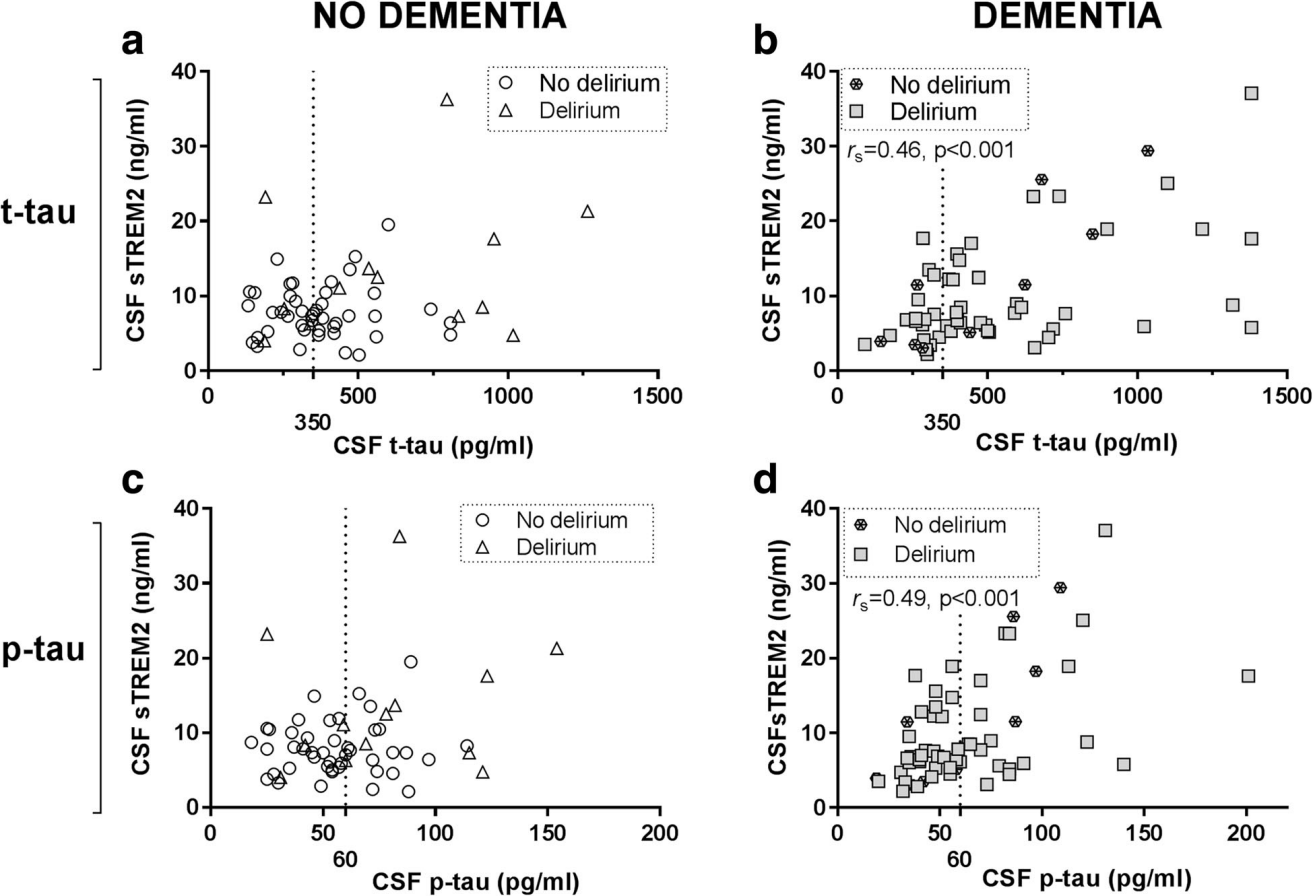
CSF levels of tau markers and sTREM2 in patients without or with pre-existing dementia. In CSF, there were no correlation between sTREM2 and either t-tau or p-tau in patients without pre-existing dementia (**a**, **c**). In contrast, both CSF tau markers, t-tau, and p-tau, correlated positively to CSF sTREM2 only in dementia patients (**b**, **d**) as indicated by significant and positive correlations (*r*_s_). The correlations were calculated for all patients with or without pre-existing dementia respectively, while patients with and without delirium are indicated by separate symbols. The dotted lines show pathological biomarker cut-off level (350 pg/ml and 60 pg/ml). CSF: cerebrospinal fluid, sTREM2: soluble TREM2, p-tau: phosphorylated_181_ -tau, t-tau: total tau, *r*_s_: Spearman’s rho

CSF t-tau and p-tau concentrations are thought to reflect altered tau metabolism that relates to neurodegeneration and tangle formation in AD [34]. CSF sTREM2 related positively to both CSF t-tau and p-tau in the entire hip fracture patient cohort (*r*_S_ = 0.32, *p* = 0.001 for both, *n* = 117). Stratification by dementia status gave results reminiscent of the CSF Aβ-data, with strong correlations to CSF sTREM2 restricted to patients with pre-existing dementia (t-tau, *r*_S_ = 0.46, *p* < 0.001; p-tau, *r*_S_ = 0.49, *p* < 0.001, *n* = 60). The relation between CSF-TREM2 and tau markers remained but were weaker when restricting the analyses to demented patients who also developed delirium (t-tau, *r*_S_ = 0.34, *p* = 0.016, *n* = 50 and p-tau, *r*_S_ = 0.37, *p* = 0.008, *n* = 50; Fig. 5, Table 2).

### CSF sTREM2 and predictive ability of delirium, age, and AD CSF-biomarker level

Only among patients with pre-existing dementia did CSF sTREM2 relate positively to the three CSF AD core biomarkers (Aβ42, t-tau, and p-tau). Here in a separate examination, CSF Aβ42 and p-tau were included in multiple linear regressions to explore if AD core biomarkers together better explained the variability of CSF sTREM2. The biomarker CSF p-tau was chosen above t-tau since it is more AD-specific [35] and since previous analyses gave slightly higher correlation coefficients to CSF sTREM2. Such analyses enabled us to examine if effects of AD neuropathology, reflected by biomarkers CSF Aβ42 and CSF p-tau, masked an effect of delirium on CSF TREM2.

Again, the hip fracture patient cohort was stratified for pre-existing dementia. Variables included were delirium, age, CSF Aβ42, and CSF p-tau. In the patient group with pre-existing dementia, the AD CSF biomarkers were included as predictors before incorporating age and delirium in the analysis. CSF p-tau explained most variability as a sole predictor of CSF TREM2 level (*R* = 0.50). Including CSF Aβ42 as a second predictor increased the explained CSF sTREM2 level variability (*R* = 0.61), while age and delirium did not further increase predictive ability (ln(CSF sTREM2): CSF p-tau *β*1 = 0.01, *p* < 0.001; CSF Aβ42 *β*2 = 0.002, *p* = 0.002; age *β*3 = 0.0001, *p* = 0.91; delirium *β*4 = − 0.03, *p* = 0.86; *n* = 59). Irrespective of the number of predictors included, the effect size (*β*) remains essentially the same (Table 3).

The patient group without pre-existing dementia was examined in the same manner with multiple linear regressions. Delirium was included as the initial predictor before incorporating age and the AD core CSF biomarkers Aβ42 and p-tau. Delirium was the only significant predictor in this patient group (*R* = 0.34 as a single predictor). CSF AD core biomarkers did not have a significant impact and their inclusion and subsequent adjustment did not influence the effect size (*β*) of delirium (ln(CSF sTREM2: delirium *β*1 = − 0.47, *p* = 0.02; CSF p-tau *β*2 = 0.000, *p* = 0.97; CSF Aβ42 *β*3 = 0.000, *p* = 0.16; age *β*4 = 0.013, *p* = 0.13, *n* = 57). This model suggest that patients without pre-existing dementia, but afflicted by a hip fracture and subsequent delirium would result in an increased CSF TREM2 level (≈ 1.5 ng/ml). This applied to conditions adjusted for interfering factors, age, or CSF biomarker levels indicating AD neuropathology (Table 3).

### CSF sTREM2 and ratios of tau and amyloid CSF biomarkers

We then examined ratios of amyloid and tau CSF biomarkers, which are more discriminative than a single biomarker to the diagnosis and prognosis of AD [36, 37]. Biomarker ratios (t-tau/Aβ42 or p- tau/Aβ42) separated non-demented patients with or without delirium (Additional file 1: Table S1), consistent with a previous report [10]. There was no correlation between CSF sTREM2 and CSF t-tau/Aβ42 and p-tau/Aβ42 among those with dementia or without dementia (Additional file 1: Table S1 and Figure S3).

### CSF sTREM2 level and AD neuropathology

AD is the major cause of dementia. To identify hip fracture patients with pre-existing dementia likely due to AD, we applied cut-off values for the CSF Aβ42 and CSF-tau [31]. Thus patients were stratified for having a CSF Aβ42-level above or below AD cut-off (Aβ−/Aβ+) and likewise tau-level (t-tau or p-tau) above or below AD cut-off (tau+/tau-) [38]. Most patients with pre-existing dementia were below CSF Aβ42 cut-off (≈ 90%), while fewer patients were above CSF-tau cut-off (≈ 40%, Table 1).

Among patients neither having delirium nor pre-existing dementia, CSF sTREM2 level did not differ between those who were above or below CSF-biomarker CSF Aβ42 level cut-off for AD (*p* = 0.22, *n* = 26 *n* = 18). Among patients without pre-existing dementia and a CSF Aβ42 level below cut-off, CSF sTREM2 was higher in those with subsequent delirium (*n* = 11) as compared to unaffected subjects (*n* = 26; *p* = 0.048). The analyses gave similar results as when all patients without pre-existing dementia were included in the analyses (*p* = 0.046, Fig. 1c). Corresponding analyses for tau (t-tau or p-tau positive) yielded essentially the same results (data not shown). These analyses confirm multiple linear regression analyses of CSF Aβ42 and CSF tau markers not influencing the CSF sTREM2 level in patients without pre-existing dementia.

### CSF sTREM2 in patients with medical delirium

**Fig. 6:**
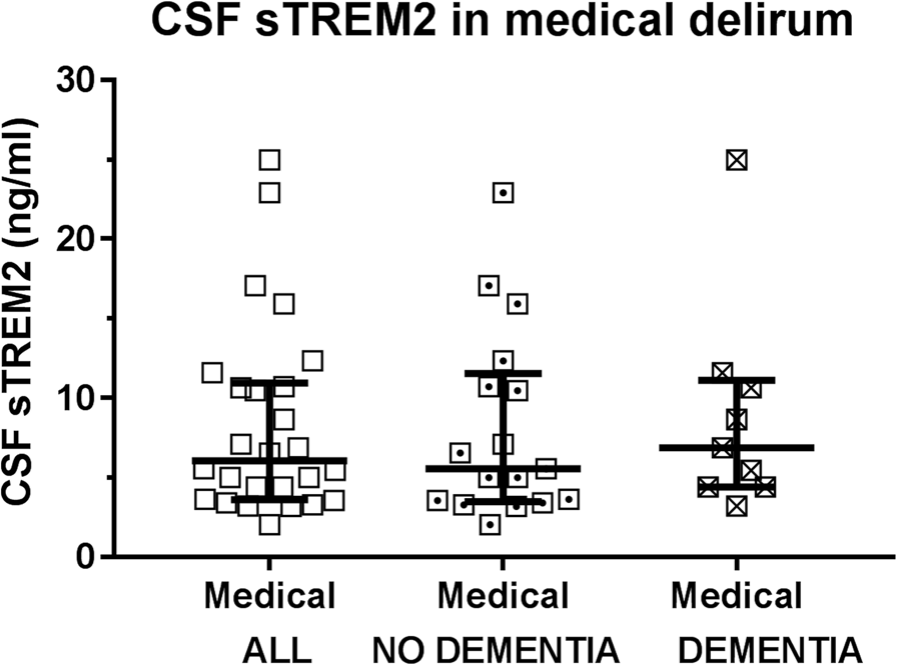
CSF sTREM2 level in patients with medical delirium. There was a tendency of a reduced CSF sTREM2 level in patients who presented with or developed delirium secondary to a medical condition compared to patients with delirium following a hip fracture (*p* = 0.08; *n* = 26, *n* = 65). Restricting the analyses to only patients without pre-existing dementia, hip fracture-triggered delirium patients displayed a higher CSF sTREM2 level than the medical delirium patients (*p* = 0.04, *n* = 15, *n* = 17). CSF: cerebrospinal fluid, sTREM2: soluble TREM2

We also included a group of delirium patients having delirium secondary to a medical condition with or without pre-existing dementia (medical delirium, *n* = 26, Table 1). There was a trend of a lower CSF sTREM2 level in patients with medical delirium as compared to those with delirium after a hip fracture (*p* = 0.08; CSF sTREM2 ng/ml: 6.1 (7.3) vs 7.7 (7.9), *n* = 26, *n* = 65, Figs. 1b and 6).

Restricting the analyses only to patients without pre-existing dementia, CSF sTREM2 was reduced in medical delirium as compared to patients with hip fracture triggered delirium (*p* = 0.04; *n* = 17, *n* = 15, Figs. 1 and 6; Table 1). Restricting the hip fracture cohort delirium patients to those with incident delirium CSF sTREM2 was lower, and close to significant (*p* = 0.055; *n* = 7, *n* = 17), but not when compared to those with prevalent delirium (*p* = 0.18, *n* = 8, *n* = 17; Figs. 2 and 6).

Analyzing only those with pre-existing dementia, CSF sTREM2 in patients with medical delirium did not statistically differ from hip fracture patients with delirium (*p* = 0.56, *n* = 9, *n* = 50; Fig. 1c and 6 and Table 1), nor when comparing to each subgroup.

### CSF sTREM2 in medical delirium patients in relation to age and gender

Like in the hip fracture cohort, we found CSF sTREM2 not related to age neither when analyzing all medical delirium patients (*r*_S_ = 0.23, *p* = 0.26, *n* = 26) nor when restricting the analysis to patients without dementia (*r*_S_ = 0.41, *p* = 0.11, *n* = 17, Additional file 1: Figure S4). Patients admitted to the hospital with medical delirium were younger with a greater age range (66(16) years, Table 1) than the patients having a hip fracture accident. When adjusting for age, the level of CSF sTREM2 was not significantly higher in hip fracture triggered delirium relative to medical delirium. However, the group size is almost too small to allow statistical age adjustment with several covariates, and the loss of significance from univariate analyses may be explained by additional noise from the added predictor (ln(CSFsTREM2) univariate model; delirium: *β*1 = 0.62, *p* = 0.05, and with a bivariate model and age included: delirium *β*1 = 0.27, *p* = 0.48, age *β*2 = 0.02, *p* = 0.15, *n* = 24).

## Discussion

This is the first study to report on CSF sTREM2 level in delirium. Moreover, the study differs from previous investigations by analyzing CSF sTREM2 as a biomarker in a dementia population of an advanced age. Although we did not see an overall effect of delirium, analyzing patients with and without pre-existing dementia separately revealed a clear differential effect on CSF sTREM2 and interrelations to other biomarkers in these two populations. Delirium increased CSF sTREM2 only in patients without pre-existing dementia. TREM2 is a highly microglial-specific receptor, and ectodomain shedding of TREM2 releases sTREM2 that then presumably drains to CSF [18]. The level of sTREM2 in the brain reflects amyloid-induced microglial activation in transgenic mice with aging as judged by PET imaging [39]. Patient studies also suggest that microglial-derived CSF sTREM2 increases with a general glial-mediated immune response, e.g., a positive relation with the astroglial CSF-marker YKL-40 [19]. We therefore argue that increased sTREM2 in delirium without pre-existing dementia is due to central microglial activation. We speculate that CSF sTREM2 increases only in delirium patients without dementia because the pathogenic process is less complex and the inflammatory process will more clearly stand out in this patient group.

Increased CSF sTREM2 with delirium triggered by hip fracture was most prominent in incident delirium, i.e., in CSF sampled before the delirium syndrome was evident in non-demented patients. Medical delirium patients all suffered encephalopathy at the time of CSF sampling. Interestingly, patients with medically induced delirium displayed lower CSF sTREM2 relative to patients with hip fracture-triggered delirium. This effect was close to significant when compared to incident delirium patients alone. Thus, stratification of delirium-afflicted patients suggests that CSF sTREM2 increases transiently prior to delirium onset, but then declines. An early but transient glial response in delirium is supported by increased levels of other CSF biomarkers of immune responses in incident delirium, such as neopterin [40] and astroglia-derived S-100β [41]. Thus, CSF sTREM2 is a promising biomarker of microglial activation that is presumably more usable to detect transient responses, which is consistent with TREM2 having a rapid cell surface turnover (< 1 h) [42]. Longitudinal studies and continuous CSF sampling of delirium patients could confirm or refute this hypothesis, although it might be ethical challenging to conduct such a study with fragile patients.

In contrast, delirium did not alter the CSF sTREM2 level in patients with pre-existing dementia nor did dementia by itself affect CSF sTREM2. CSF sTREM2 is reported increased in younger and CSF biomarker-selected AD patients (mean ≈ 65–70 years) (e.g., [19, 20]). We speculate that superimposed dementia with Aβ deposits and tau inclusions continuously stimulating microglial activation diminish the effect of delirium on microglial response and sTREM2 release. Such proteinaceous neuropathology might also lead to an increased protein turnover preventing a raised interstitial sTREM2 level in demented patients. Importantly, as compared to many other CSF-dementia studies, the patients examined by us were markedly older (median ≈ 85 years) and not biomarker-selected and their dementia pathogenesis was likely more heterogeneous [43, 44]. Thus, comorbidities and advanced age could have diluted the direct link from AD neuropathology to enhanced sTREM2 release and CSF sTREM2.

The initial hip fracture trauma presumably activated peripheral immune responses in the patients. The neuroinflammatory hypothesis of delirium suggests that brain dysfunctions and clinical presentation are secondary to peripheral immune activation [4]. Dementia patients displaying increased CSF sTREM2 with waiting time for acute hip fracture surgery is consistent with this theory. That this only was evident among dementia patients also support the concept of primed more easily activated microglia in neurodegenerative disease compared to a healthy brain [45, 46].

Exploring relations between CSF biomarkers may provide better pathogenic understanding by hinting to simultaneously ongoing processes in the brain. CSF Aβ and t-tau/p-tau markers related positively to CSF sTREM2 only in dementia patients with delirium, possibly suggesting somewhat distinct biological processes of delirium with or without pre-existing dementia. CSF sTREM2 related positively to CSF Aβ42 in the demented patients, essentially all of which were below the CSF Aβ42 cut-off level. Clinical data suggest that Aβ42 is sequestered by senile plaques, long before symptom onset leading to a pronounced drop in CSF Aβ42. This low CSF Aβ42 level is then stable in the individual patient [47, 48]. Thus, a positive relation between CSF Aβ42 and CSF sTREM2 in demented patients does presumably not relate to the extent of amyloid deposition. Instead, it is more likely to reflect shared protein synthesis and metabolism of Aβ precursor protein (AβPP) and TREM2, an idea which is consistent with our findings of positive relations also to the shorter and far less plaque-sequestered C-terminal truncated peptides Aβ38 and Aβ40 [49]. AβPP and TREM2 share common features of ectodomain shedding by ADAMs α-secretase and subsequent γ-secretase cleavage [18, 50–52], and although they are released by different cell types, one can speculate whether both play a role to similar physiological functions that involve neuronal-glial communication.

An expanding literature links Aβ production to neuronal and synaptic activity and biological rhythms, e.g., sleep-wakefulness [53, 54]. We speculate that delirium in dementia patients triggers neuronal network activation with concomitant enhanced release of Aβ peptides, t-tau/p-tau, and sTREM2; all reflected by a transiently increased CSF level. Interestingly, a recent study demonstrated with isotope labeling kinetics that increased CSF-tau in early AD reflects neuronal tau-synthesis and positively correlates with amyloid-PET, but not tau-PET [55]. Thus, CSF- t-tau/p-tau levels might also reflect neuronal activity associated with amyloid, and not simply axonal damage and tauopathy as previously thought. There are reports of neuronal network disturbances in Alzheimer’s disease as well as delirium [56, 57]. Thus, our observations of positive CSF-biomarker associations only in demented patients might be due to the susceptibility of frail brains to neuronal network dysfunctions.

CSF sTREM2 and tau markers interrelated in several studies of AD cohorts, yet in different subpopulations [19, 20, 58]. CSF sTREM2 clearly increased in a cohort of suspected non-amyloid pathology (SNAP) patients having cognitive dysfunctions and only positive CSF-tau biomarkers indicating that elevated CSF sTREM2 can occur independent of amyloid pathology [58]. Indeed, inclusion of p-tau in linear regression analyses showed that p-tau was the best sole predictor. Including CSF Aβ42 as a second predictor better explained CSF sTREM2 variability in the dementia group, while incorporating variables age and delirium did not. Thus, in dementia patients, the existing neuropathology seemed to exceed any delirium effects again suggesting pathogenic differences of delirium with and without pre-existing dementia.

With aging, dementia does not well relate to Aβ pathology and tauopathy, presumably because of increased importance of cerebrovascular comorbidity [59]. The high median age and limited age distribution of the study cohort likely explain why CSF sTREM2 did not relate to age. There might also be a ceiling effect of CSF sTREM2 with aging, which would be worth further studying. CSF sTREM2 increases with aging in several studies that involved younger patient populations [20–22]. Indeed, there was a tendency of a positive correlation with aging among patients with medical delirium that included younger individuals.

## Conclusion

The present biomarker analyses hint at the biological processes of delirium being dependent on pre-existing dementia with selectively increased CSF sTREM2 in delirium only in the absence of dementia. These preliminary findings need replication in independent patient cohorts and assays. Statistical power is a study limitation with small cohorts after stratification. CSF sampling aged frail patients in a confused state is practically and ethically difficult and the CSF material used is unique in size and composition. As judged by mutant TREM2-variants, both increased and decreased shedding can reflect reduced cell surface TREM2 and impaired gene function [51, 60]. Increased sTREM2 likely represents microglial activation but whether such central immune activation is beneficial or detrimental to brain functions remains unclear. Increased CSF sTREM2 prior to delirium could possibly serve as a delirium risk indicator, albeit not alone. An inflammatory CSF biomarker panel that includes sTREM2 might aid identification of at-risk patients with special care requirements in the post-operative care unit.

## Additional file

Additional file 1: **Figure S1.** CSF sTREM2 and the relation to age in hip fracture patients. **Figure S2.** Triplex MSD measurements of CSF Aβ peptides in dementia patients. **Table S1.** CSF t-tau/Aβ42 and p-tau/Aβ42 ratios and correlations to CSF sTREM2 in the hip fracture cohort. **Figure S3.** CSF sTREM2 and the ratios of t-tau/Aβ42 and p-tau/Aβ42. **Figure S4.** CSF sTREM2 and age in medical delirium patients (PDF 4106 kb)

## Abbreviations

AD: Alzheimer’s disease
ADAMs: A disintegrin and metalloprotease
Aβ38: Amyloid beta 1–38
Aβ40: Amyloid beta 1–40
Aβ42: Amyloid beta 1–42
AβPP: Aβ precursor protein
CAM: Confusion Assessment Method
CNS: Central nervous system
CSF: Cerebrospinal fluid
ELISA: Enzyme-linked immunosorbent assay
ln: Natural logarithm
LP: Lumbar puncture
MCI: Mild cognitive impairment
OUS: Oslo University Hospital
p-tau: Phosphorylated tau
sTREM2: Soluble TREM2
TREM2: Triggering receptor expressed on myeloid cells 2
t-tau: Total tau
