# Supplementary material for: CSF sTREM2 in delirium—relation to Alzheimer’s disease CSF biomarkers Aβ42, t-tau and p-tau
Source: J Neuroinflammation. 2018 Nov 3;15:304. doi: 10.1186/s12974-018-1331-1 (PMC6215363; doi:10.1186/s12974-018-1331-1)
Supplement: Supplementary file 1 — Figure S1. CSF sTREM2 and the relation to age in hip fracture patients. Figure S2. Triplex MSD measurements of CSF Aβ peptides in dementia patients. Table S1. CSF t-tau/Aβ42 and p-tau/Aβ42 ratios and correlations to CSF sTREM2 in the hip fracture cohort. Figure S3. CSF sTREM2 and the ratios of t-tau/Aβ42 and p-tau/Aβ42. Figure S4. CSF sTREM2 and age in medical delirium patients (PDF 4106 kb) [file 12974_2018_1331_MOESM1_ESM.pdf]

SUPPLEMENTARY MATERIAL

CSF sTREM2 in delirium - relation to Alzheimer's disease CSF biomarkers AB42, t-tau and p-tau

Henjum K, Quist-Paulsen, Zetterberg H, Blennow K, Nilsson LNG, Watne LO,

FIG S1 CSF sTREM2 and the relation to age in hip fracture patients

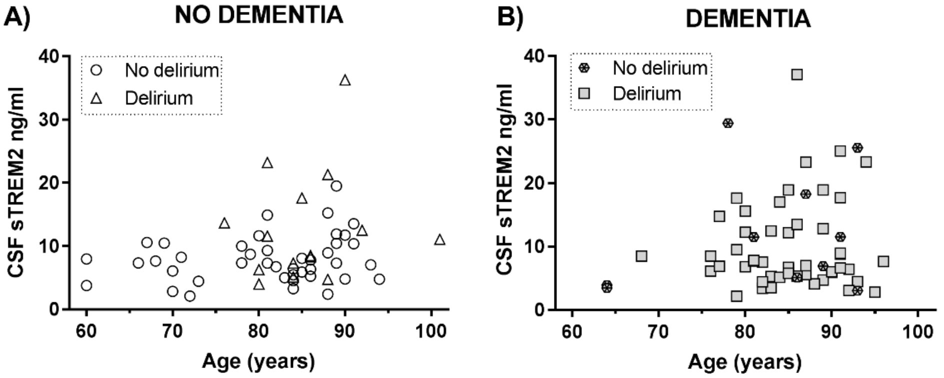

Fig. S1. CSF sTREM2 in relation to age among patients in the hip fracture cohort. (A) There was no relation between age and sTREM2 among those without pre-existing dementia (r_s =0.21, p=0.12, n=59). (B) Likewise age and sTREM2 did not relate among patients with pre-existing dementia (r_s=-0.003, p=0.98, n=61). Patients with and without delirium are indicated by separate symbols, but the correlations are presented for all patients with or without pre-existing dementia. CSF: Cerebrospinal fluid, sTREM2: soluble TREM2. r_S: Spearman's Rho.

FIG S2: Triplex MSD-measurements of CSF Aβ-peptides in dementia patients

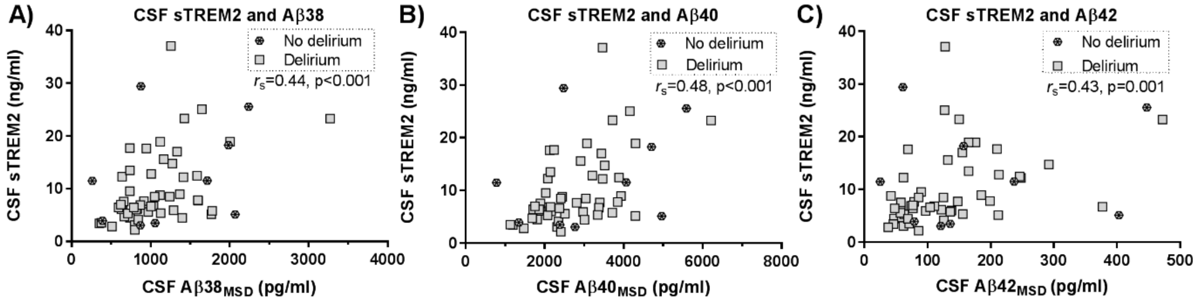

Fig. S2. The CSF sTREM2 level correlated positively with CSF Aβ mesoscale measurements in patients with pre-existing dementia. (A) CSF Aβ1-38, (B) CSF Aβ1-40 and (C) CSF Aβ1-42 in relation to CSF sTREM2. The correlations were calculated for all dementia patients. Patients with and without delirium are indicated by separate symbols. CSF: Cerebrospinal fluid, sTREM2: soluble TREM2. r_S: Spearman's Rho.

TABLE S1: CSF t-tau/Aβ42 and p-tau/Aβ42 ratios and correlations to CSF sTREM2 in the hip fracture cohort

	PATIENTS *WITHOUT* DEMENTIA				PATIENTS *WITH* DEMENITA			
	**All**	**No Delirium**	**Delirium**	**p**	**All**	**No Delirium**	**Delirium**	**p**
Median (ICR), *N*	*59*	*44*	*15*		*60*	*9*	*50*	
CSF t-tau/Aβ42	0.75 (1.04)	0.62 (0.64)	1.92 (2.32)	<0.001	1.55 (1.73)	1.08 (1.92)	1.55 (1.77)	0.38
CSF p-tau/Aβ42	0.11(0.10)	0.10 (0.08)	0.27 (0.26)	0.001	0.21 (0.20)	0.14 (0.21)	0.21 (0.19)	0.45
CSF sTREM2 correlations, *N*	*57*	*44*	*13*		*60*	*9*	*50*	
CSF t-tau/Aβ42	0.08 (p=0.53)	-0.10 (p=0.57)	N.A		0.12 (p=0.36)	N.A	-0.04 (p=0.78)	
CSF p-tau/Aβ42	0.02 (p=0.89)	-0.16 (p=0.31)	N.A		0.05 (p=0.70)	N.A	-0.13 (p=0.37)	

Table S1: P-values (p) of difference between patients with or without delirium are two-tailed obtained with Mann-Whitney test. Both ratios differed between patients with and without dementia (p<0.001). Correlations are presented as Spearman's Rho (p-value). N.A. not applicable due limited group size.

FIG S3: CSF sTREM2 and the ratios of t-tau/Aβ42 and p-tau/Aβ42

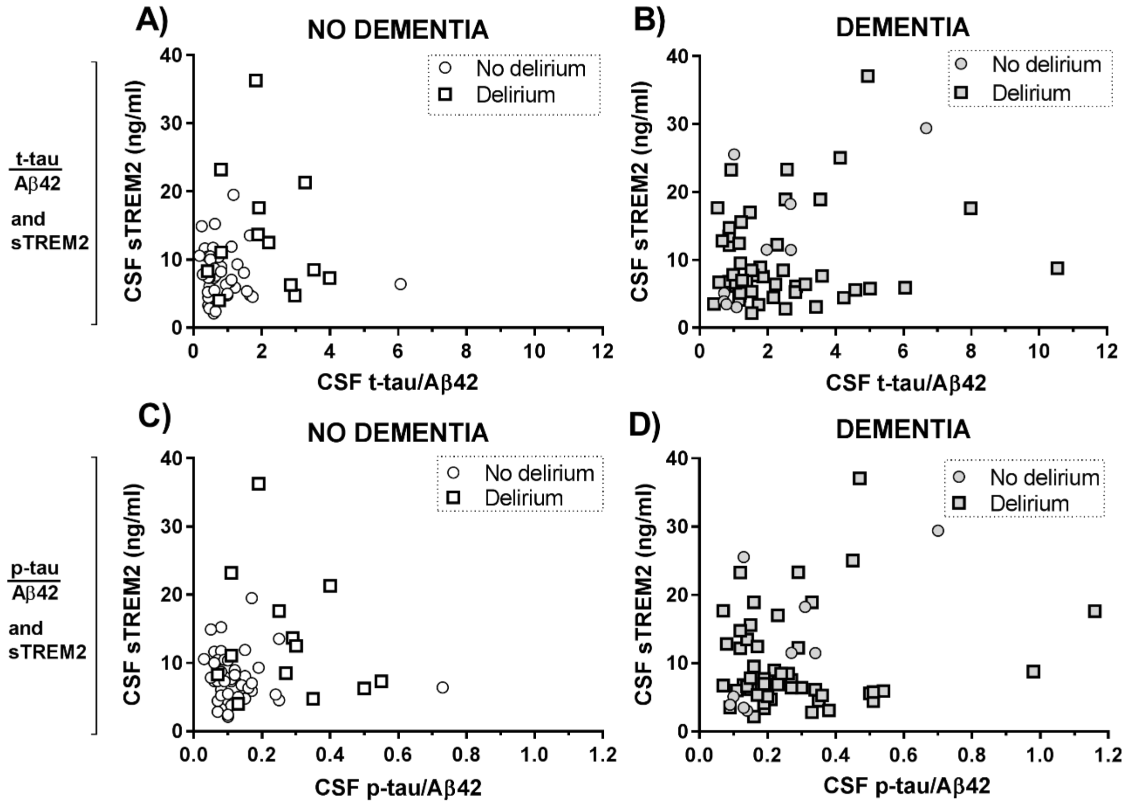

Fig S3 CSF sTREM2 did not correlate with the ratios of t-tau/Aβ42 or p- tau/Aβ42 in the hip fracture cohort. (A) There was neither a correlation between t-tau/Aβ42 and sTREM2 among those without pre-existing dementia (r_s =0.08, p=0.53, n=59) nor (B) among patients with pre-existing dementia (r_s=-0.12, p=0.36, n=60). Likewise, for p-tau/ Aβ42 (C) there was no correlation between p-tau/Aβ42 and sTREM2 among those without pre-existing dementia (r_s =0.02, p=0.89, n=59) nor (D) among patients with pre-existing dementia (r_s=-0.05 p=0.70, n=60). Patients with and without delirium are indicated by separate symbols, but the correlations are presented for all patients with or without pre-existing dementia. CSF: Cerebrospinal fluid, sTREM2: soluble TREM2. r_s: Spearman's Rho

FIG S4: CSF sTREM2 and age in medical delirium patients

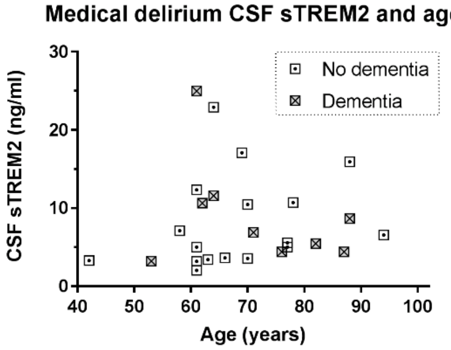

Fig S4. CSF sTREM2 in relation to age among patients with medical delirium. There was no statistical significant correlation between sTREM2 and age in medical delirium patients when analyzed by Spearman's correlation coefficient (r_s=0.23, p=0.26, n=26). Excluding patients with pre-existing dementia did increase the correlation yet it did not reach statistical significance (r_s=0.41, p=0.11, n=17). CSF: Cerebrospinal fluid, sTREM2: soluble TREM2. r_s: Spearman's Rho.
